# Comparison of the Natural Vibration Frequencies of Timoshenko and Bernoulli Periodic Beams

**DOI:** 10.3390/ma14247628

**Published:** 2021-12-11

**Authors:** Łukasz Domagalski

**Affiliations:** Faculty of Civil Engineering, Architecture and Environmental Engineering, Łódź University of Technology, 90-924 Lodz, Poland; lukasz.domagalski@p.lodz.pl

**Keywords:** periodic beam, Timoshenko beam, tolerance modelling, vibrations

## Abstract

This paper deals with the linear natural vibrations analysis of beams where the geometric and material properties vary periodically along the beam axis. In contrast with homogeneous prismatic beams, the frequency spectra of such beams are irregular as there exist enlarged intervals between some adjacent frequencies. Presented here are two averaged models of beams based on the tolerance modelling approach. The assumptions of classical Euler–Bernoulli and Timoshenko–Ehrenfest beam theories are adopted as the foundations. The resulting mathematical models are systems of differential equations with constant, weight-averaged coefficients. This makes it possible to apply any classical method of solution suitable for homogeneous beams, such as Galerkin orthogonalization. Here, emphasis is placed on the comparison of natural frequencies neighbouring the frequency band-gaps that are obtained from these two theories. Two basic cases of material and geometric property distribution in a periodicity cell are studied, and the natural frequencies and mode shapes are obtained for a simply supported beam. The results are supported by a comparison with the finite element method and partially exact solutions.

## 1. Introduction

Here, the considered periodic beams consist of repeated identical segments made from two or more different materials or segments made of a single material but with a variable cross-section dimensions or shape. Such structural elements possess certain distinguishing dynamic features, such as the existence of gaps in the frequency spectrum. The very mechanism of the gap occurrence in finite beams is clear. It is caused by the periodic distribution of the stiffness and mass properties that tends to favour certain vibration shapes and, as a result, the corresponding frequencies.

More precisely, as every mode shape consists of a finite number of half-waves, it is important if the mode shape nodes or antinodes are close or overlap with maximum or minimum stiffness and mass points. As a consequence, the frequency gaps for a simply supported beam are expected near frequencies for which the corresponding number of half-waves *n* is an even multiple or divisor of number of cells L/l (for an even number of cells) or L/l±1 (for an odd number of cells).

This paper focuses on linear-elastic beams with a periodic structure considered in the framework of the Euler–Bernoulli (abbreviated: Bernoulli) and Timoshenko–Ehrenfest (abbreviated: Timoshenko) theories. Generally speaking, the former of these theories has its application limited to analysis of slender beams, as it is a special case of the latter. The second theory takes into account both shear deformations and rotatory inertia effects, which makes it suitable for the analysis of moderately thick beams.

Many interesting remarks about these theories and their applications in the analysis of stepped beams can be found in the works [1,2,3,4,5,6], the first of which draws attention to the authorship of the so-called Timoshenko theory. There are many engineering fields in which multiple-stepped beams can be applied.

Structures, such as aircraft wings, long span truss and beam bridges, suspended pipelines and rotatory unbalanced shafts can be approximately represented as such. For example, high buildings made from repetitive floors can be modelled as clamped beams with effective cross-section and equidistant lumped masses. There are other problems, such as periodically damaged structures, e.g., cracked reinforced concrete beams. In many of these domains, high-frequency vibrations are of crucial importance. Future applications are in the fields of vibration control and acoustic insulation.

Thus, it is desirable to develop efficient methods to make the analysis of low and high frequencies of these structures possible. The question arises: can an averaged model yield correct results for such non-continuous structures? The answer to this question can be pursued in various ways, including through numerical experiments as presented in this paper.

Dynamical problems of beams with periodic distributions of material and geometric properties are governed by differential equations with highly oscillating, often non-continuous coefficients. There are various ways to tackle this problem. One group of these methods is based on discretization by means of the Finite Element Method, Finite Difference Method, or others. The second group strives to obtain simplified models, often based on homogenization. The resulting models replace the original, non-homogeneous structure with an equivalent homogeneous one with effective constant or slowly varying properties.

Among them, the theory of asymptotic homogenization [7] is one of the most popularized due to its mathematical rigorousness. However, these models often neglect the effect of the periodicity cell size, which is sufficient in the static analysis of such structures. In this paper, the tolerance-averaging technique (TA) [8] is applied in order to obtain averaged, non-asymptotic models of periodic beams. The resulting differential equations have constant, weight-averaged coefficients, some of which are dependent on the periodicity cell dimension.

In [9], Timoshenko’s composite beam functions and Mindlin–Reissner thick plate theory were applied to a non-linear FE analysis of thin to moderately thick plates and slabs made of reinforced concrete. Non-classical, microstructure dependent Timoshenko beam models were analysed in [10,11,12], respectively. The effects of the moving load, boundary condition and material gradation of a bi-directional functionally graded beam on free and forced vibrations were examined in the framework of thin and thick beams in [13].

Periodically supported Timoshenko beams were analysed in [14]. Dynamic problems of periodic Timoshenko beams resting on a two-parameter elastic foundation were investigated in [15] by means of the weak-form quadrature element method. In [16], the problem of wave propagation in periodic Timoshenko beams on elastic foundations under moving loads, taking into account tensile and compressive axial load, was analysed.

Theoretical and experimental analysis of flexural vibration band gaps in Timoshenko beams with locally resonant structures was presented in [17]. In [18], the third order shear deformation theory was applied in the analysis of free vibrations of two directional functionally graded beams, taking into account various sets of boundary conditions. Non-linear vibrations of Timoshenko beams using FEM were investigated in [19].

The cell-centre finite volume method was applied in the analysis of static and natural non-linear vibration analysis of functionally graded beams in [20]. Composites made of an isotropic elastic matrix that contain periodically placed inclusions or voids were analysed in [21].

A method of obtaining specified or extreme effective stiffness via topology optimization of the microstructure design was developed in [22]. Vibrations of composite structures, according to various beam theories, were considered in several papers. The beam can be modelled as a Timoshenko beam [23,24], Rayleigh beam [25,26] or Euler–Bernoulli beam [27].

Dynamic analysis can be extended based on the Winkler model [28,29], Pasternak model, a combination of both (the Winkler–Pasternak model), a nonlinear elastic model and fractional order viscoelastic model [30].

Researchers have presented methods to analyse structures resting on elastic foundations. The stability of periodic shells on elastic foundations subjected to external loading was considered in [31]. Papers [31,32] investigated the periodic behaviour of functionally graded plates and shells, respectively. Thin walled beams were analysed in [33], whereas the stress distributions and capabilities through a simple numerical example were demonstrated.

A theoretical study on the propagation of the flexural wave in the periodic beam on elastic foundation was presented in [34]. Moreover, the waves propagation was theoretically and experimentally investigated in straight beams with a periodic structure [35].

The tolerance-averaging technique was used for the static analysis [36] and various dynamic problems, with reference in particular, to beams [37,38], plates [39], shells [40,41,42,43] and thin-walled structures [44,45].

The aforementioned method was also applied to thermal problems [46,47,48] of periodic laminates, longitudinally graded materials, micro-heterogeneous hollow cylinders and cylindrical composite conductors, respectively. This work is a follow-up of earlier papers published by the author, together with other participants, on vibrations of periodic beams [49,50].

Although the results provided in these papers were satisfactory to some extent, the theory has been improved since then, mainly due to the use of a new class of weakly slowly varying functions in the analysis of shear-deformable beams. This contributed to an increase in the scope of application of the proposed models. An attempt was also made to compare the results of the proposed model with the exact results.

As stated before, vibration analysis of periodic beam type structures has recently received a remarkable amount of attention due to the importance and various applications of the subject. The tolerance-averaging approach is an effective analytical-numerical technique for problems that concern periodic structures. The main aim of this paper is to compare natural vibration frequencies and mode shapes of Bernoulli and Timoshenko beams using this approach.

The paper is outlined as follows. The theoretical background of the Bernoulli and Timoshenko beams is contained in Section 2. The tolerance approach and its fundamental assumptions are introduced and briefly discussed in Section 3. A brief derivation of the model equations in terms of both theories is presented in Section 4. In Section 5, the considered calculational problem is stated, and the solution methodology is described. In Section 6, the results and discussion are provided. The paper ends with our general conclusions.

## 2. Formulation of the Problem

**Figure 1:**
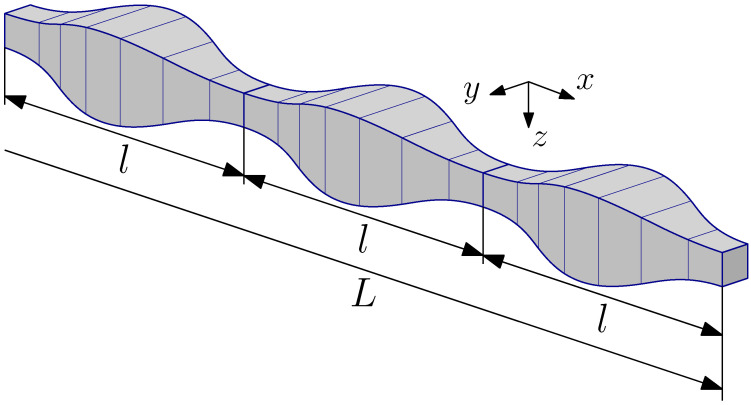
A fragment of a periodic beam.

We considered a beam made of linearly elastic material. The geometry of the beam is described in an orthogonal Cartesian coordinate system Oxyz. The Ox axis coincides with the axis of the beam, Figure 1. The inertial forces act in the direction of the axis Oz, and the Oxz plane of the load is, at the same time, the symmetry plane of the beam cross-section. With these assumptions, the problem becomes one-dimensional.

The region occupied by the beam is defined as Ω ≡ [0, *L*], where *L* stands for the beam length. We assume that the beam consists of many repetitive small elements, called periodicity cells. The periodicity cell is defined as $\Delta \;\equiv \;\left[-l/2,l/2\right]$, where l≪L is the length of the cell. It is assumed that the beam thickness is of the order of the inhomogeneity period and that the deflections are small compared to the beam thickness.

### 2.1. Governing Equations of Euler–Bernoulli Beam Theory

The Euler–Bernoulli beam theory assumes that the sections perpendicular to an undeformed beam axis remain planar and normal to the axis after deformation. This theory also neglects the effect of rotational inertia. Let $w=w\left(x,t\right)$ be the transverse deflection, $EJ=E\left(x\right)J\left(x\right)$ be flexural stiffness, $\mu =\rho \left(x\right)A\left(x\right)$ be mass per unit length. Let $\partial^{k}=\partial^{k}/\partial x^{k}$ be the *k*-th derivative of a function with respect to the *x*-coordinate; overdot stands for the derivative with respect to time.

The strain–displacement relations are assumed as follows:

(1) $$\kappa =\partial^{2}w,$$

where κ stands for the beam axis curvature. Then, the relations between stress resultants (internal forces) and displacements can be introduced:

(2) $$\begin{array}{cc} \; & M=EJ\kappa =EJ\partial^{2}w, \end{array}$$

where *M* is the bending moment.

The strain and kinetic energy density per unit length of the beam are given by:

(3) $$W=\frac{1}{2}\left(M\kappa \right),\;K=\frac{1}{2}\left(\mu \dot{w}\dot{w}\right).$$

The equations of motion can be obtained from the principle of stationary action A, formulated as:

(4) $$\delta A=\delta \int_{t_{0}}^{t_{1}}\int_{0}^{L}Ldxdt=\int_{t_{0}}^{t_{1}}\int_{0}^{L}\delta Ldxdt=0,$$

cf. [8], where the Lagrangian $L=L\left(x,t,w,\partial^{2}w,\dot{w}\right)$ is given by the formula:

(5) L=W−K,

and since the variations of unknown functions have to vanish at $t=t_{0},t_{1}$, Equation (4) can be rewritten as:

(6) $$\begin{array}{cc} \delta A & =\int_{t_{0}}^{t_{1}}\int_{0}^{L}\left[M\delta \kappa +\mu \ddot{w}\delta w\right]dxdt=0. \end{array}$$

The differential equation of motion resulting from the above assumptions can be written as:

(7) $$\begin{array}{cc} \; & \partial^{2}\left(EJ\partial^{2}w\right)+\mu \ddot{w}=0. \end{array}$$

### 2.2. Governing Equations of Timoshenko Beam Theory

The Timoshenko beam theory includes both shear deformation and rotational inertia effects. This makes the theory applicable in the analysis of thick beams, sandwich composite beams, or beams subjected to high-frequency excitation when the wavelength is on the order of the beam thickness. In addition to the previous section, we introduce some new denotations: let $\theta =\theta \left(x,t\right)$ be the average cross-section rotation and $\gamma =\gamma \left(x,t\right)$ be the shear angle. Let $kGA=k\left(x\right)G\left(x\right)A\left(x\right)$ be the shear stiffness and $\vartheta =\rho \left(x\right)J\left(x\right)$ be the rotational moment of inertia per unit length.

The strain–displacement relations are assumed as follows:

(8) κ=∂θ,γ=∂w−θ,

as the cross-section rotation is no longer assumed to be equal to the deflection slope. Then, the relations between stress resultants (internal forces) and displacements can be introduced:

(9) $$\begin{array}{c} \begin{array}{cc} \; & M=EJ\kappa =EJ\partial \theta ,\\ \; & Q=kGA\gamma =kGA\left(\partial w-\theta \right) \end{array} \end{array}$$

where *M*, and *Q* are the bending moment and shear force, respectively.

The strain and kinetic energy density per unit length of the beam can then written as:

(10) $$W=\frac{1}{2}\left(M\kappa +Q\gamma \right),\;K=\frac{1}{2}\left(\mu \dot{w}\dot{w}+\vartheta \dot{\theta }\dot{\theta }\right).$$

Again, equations of motion can be obtained from the principle of stationary action A (4); however, now, the Lagrangian (5) is a function of different arguments, $L=L\left(x,t,w,\theta ,\partial w,\partial \theta ,\dot{w},\dot{\theta }\right)$, and we obtain:

(11) $$\delta A=\int_{t_{0}}^{t_{1}}\int_{0}^{L}\left[M\delta \kappa +Q\delta \gamma +\mu \ddot{w}\delta w+\vartheta \partial \ddot{w}\partial \delta w\right]dxdt=0.$$

The system of coupled differential equations of motion resulting from the above assumptions can be written as:

(12) $$\begin{array}{cc} \; & \partial \left[kGA\left(\partial w-\theta \right)\right]-\mu \ddot{w}=0,\\ \; & \partial \left(EJ\partial \theta \right)+kGA\left(\partial w-\theta \right)-\vartheta \ddot{\theta }=0. \end{array}$$

The coefficients of Equations (6) and (12) are highly oscillating, often non-continuous functions of the *x*-coordinate. Subsequently, we present two averaged models of beams in order to circumvent this drawback.

## 3. Introductory Concepts and Basic Assumptions of the Tolerance Modelling

The tolerance modelling (or tolerance-averaging) technique (TA), cf. [8] is based on a set of intuitive heuristic concepts, such as tolerance relations, slowly varying functions and fluctuation shape functions. The most important of them are described here.

The operation of averaging over a region of periodicity cell is defined as:

(13) $$\langle f\rangle \left(x\right)\equiv \frac{1}{\left|\Delta \right|}\int_{\Delta \left(x\right)}f\left(y\right)dy,\;y\in \Delta \left(x\right),x\in \Omega_{\Delta },$$

where y are coordinates in the local system associated with the cell, $\Omega_{\Delta }$ is the region containing cell centres.

Let Ω be a regular region in $R^{m}$ and λ be a positive real number. Points $x=\left(x_{1,}\ldots ,x_{m}\right)$ and $y=\left(y_{1,}\ldots ,y_{m}\right)$ that belong to Ω are in tolerance determined by λ if the distance between those points is equal or less than λ. Now, let δ be a positive number. Real numbers μ, ν are said to be in tolerance determined by δ if the absolute value of the difference between these numbers does not exceed δ. These relations can be written as:

(14) $$\begin{array}{cc} \left(i\right)\; & x\overset{\lambda }{\approx }y\Leftrightarrow {∥x-y∥}_{R^{m}}\leq \lambda ,\\ \left(ii\right)\; & \mu \overset{\delta }{\approx }\nu \Leftrightarrow \left|\mu -\nu \right|\leq \delta . \end{array}$$

A function $F\left(\cdot \right)$ will be called slowly varying, $F\in SV_{\delta }^{R}\left(\Omega ,\Delta \right)$, of the *R*-th kind with respect to cell Δ and the set of tolerance parameters $\delta =\left(\lambda ,\delta_{0},\delta_{1},\ldots ,\delta_{m}\right)$ if and only if the following conditions are satisfied:

(15) $$\begin{array}{cc} \left(i\right) & \;\left(\forall \left(x,y\right)\in \Omega^{2}\right)\left[\left(x\overset{\lambda }{\approx }y\right)\Rightarrow F\left(x\right)\overset{\delta_{0}}{\approx }F\left(y\right)\;\mathrm{and}\;\partial^{k}F\left(x\right)\overset{\delta_{0}}{\approx }\partial^{k}F\left(y\right),\;k=1,2,\ldots ,R\right],\\ \left(ii\right) & \;\left(\forall x\in \Omega \right)\left[\lambda {\left|\partial^{i}\left(\partial^{\left(k-1\right)}F\left(x\right)\right)\right|}^{\delta_{\left(k-1\right)}}\approx 0,\;i=1,\ldots ,m\;k=1,2,\ldots ,R\right]. \end{array}$$

A function $F\left(\cdot \right)$ will be referred to as weakly slowly varying, $F\in WSV_{\delta }^{R}\left(\Omega ,\Delta \right)$, if only the first of the above conditions is satisfied. The tolerance parameter λ is known a priori as a diagonal of the periodicity cell, and the $\delta_{k}$ parameters can be determined a posteriori. Let $h\left(\cdot \right)$ be a λ-periodic highly oscillating function defined in Ω and on its boundary, continuous together with its gradients $\partial^{k}h$, k=1,…,R−1, and let it have piecewise continuous bounded gradient $\partial^{R}h$. The function is called the fluctuation shape function of the *R*-th kind $h\in FS{}_{\delta }^{R}\left(\Omega ,\Delta \right)$, if it depends on λ as a parameter and satisfies the following conditions:

(16) $$\begin{array}{cc} \left(i\right)\; & h\in O\left(\lambda^{R}\right),\;\partial^{k}h\in O\left(\lambda^{R-k}\right),\;k=1,2,\ldots ,R,\\ \left(ii\right)\; & \int_{\Delta \left(x\right)}\mu \left(y\right)h\left(y\right)dy=0,\;y\in \Delta \left(x\right),x\in \Omega_{\Delta }, \end{array}$$

for $\mu \left(\cdot \right)$ being a certain positive valued λ-periodic function defined in Ω.

The tolerance-averaging approximation will be introduced only for the cases applicable in this paper. Let $b\left(\cdot \right)$, $c\left(\cdot \right)$ be arbitrary integrable λ-periodic functions (which usually are the medium physical properties) defined in Ω, and let us introduce the functions:

(17) $$\begin{array}{cc} h\left(\cdot \right) & \in FS{}_{\delta }^{C}\left(\Omega ,\Delta \right),\\ F\left(\cdot \right) & \in WSV{}_{\delta }^{C}\left(\Omega ,\Delta \right), \end{array}$$

For example, for C=1, the tolerance approximations have the form:

(18) $$\begin{array}{cc} \langle bF+c\partial F\rangle \left(x\right) & ={\langle bF+c\partial F\rangle }_{T}\left(x\right)\equiv \langle b\rangle F\left(x\right)+\langle c\rangle \partial F\left(x\right)+O\left(\lambda \right),\\ \langle bhF+c\partial \left(hF\right)\rangle \left(x\right) & ={\langle bhF+c\partial \left(hF\right)\rangle }_{T}\left(x\right)\equiv \langle bh\rangle F\left(x\right)+\langle c\partial h\rangle F\left(x\right)+\langle ch\rangle \partial F\left(x\right)+O\left(\lambda \right), \end{array}$$

and the terms $O\left(\lambda \right)$ are negligible.

The micro–macro decomposition is based on the observation than the response of a periodic structure is periodic-like (periodic with respect to cell Δ and tolerance parameters). Let us then decompose the transverse deflection and cross section rotation angle into their slowly varying and tolerance periodic parts (summation convention holds for superscript indices):

(19) $$\begin{array}{cc} w\left(x,t\right) & =W\left(x,t\right)+h^{A}V^{A}\left(x,t\right),\;A=1,\ldots ,N,\\ \theta \left(x,t\right) & =\Theta \left(x,t\right)+p^{R}Z^{R}\left(x,t\right),\;R=1,\ldots ,N, \end{array}$$

where the decomposition of w(x,t) is valid for Bernoulli beam theory only, and decompositions of both w(x,t) and θ(x,t) are valid for Timoshenko beam theory. The new unknowns—the averaged transverse deflection, cross-section rotation and their fluctuation amplitudes—are weakly slowly varying functions of the *C* kind, respectively:

(20) $$W\left(\cdot \right),\;V^{A}\left(\cdot \right),\;\Theta \left(\cdot \right),\;Z^{R}\left(\cdot \right)\in WSV_{\delta }^{C}\left(\Omega ,\Delta \right),$$

and the corresponding fluctuation shape functions (*FS*s) are λ-periodic highly oscillating functions:

(21) $$h^{A}\left(\cdot \right),p^{R}\left(\cdot \right)\in FS{}_{\delta }^{C}\left(\Omega ,\Delta \right),$$

which should approximate the deviation of the displacements in a cell from the average motion caused by the periodic structure. In the above relations, values C=1,2 define the classes of the unknown functions that are determined for the Timoshenko and Bernoulli beam theories, respectively.

## 4. Averaged Models

After substitution the micro–macro decompositions (19) into the Lagrangian (5), the next step of modelling is averaging (13) over an arbitrary periodic cell with approximations (18). The variations of the unknown functions are as follows:

(22) $$\delta w=\delta W+h^{A}\delta V^{A},\;\delta \theta =\delta \Theta +p^{R}\delta Z^{R}.$$

Now, the variation of the averaged action functional has the following form:

(23) $$\delta A_{h}=\delta \int_{t_{0}}^{t_{1}}\int_{0}^{L}\langle L_{h}\rangle dxdt=\int_{t_{0}}^{t_{1}}\int_{0}^{L}\delta \langle L_{h}\rangle dxdt=0.$$

### 4.1. Timoshenko Beam Tolerance Model

Let us now introduce tolerance-averaged bending moments and shear forces for the Timoshenko beam model:

(24) $$\left\{\begin{array}{c} \langle M\rangle \\ \langle M\partial p^{R}\rangle \\ \langle Mp^{R}\rangle \end{array}\right\}=\left[\begin{array}{ccc} \langle EJ\rangle & \langle EJ\partial p^{S}\rangle & \langle EJp^{S}\rangle \\ \langle EJ\partial p^{R}\rangle & \langle EJ\partial p^{R}\partial p^{S}\rangle & \langle EJ\partial p^{R}p^{S}\rangle \\ \langle EJp^{R}\rangle & \langle EJp^{R}\partial p^{S}\rangle & \langle EJp^{R}p^{S}\rangle \end{array}\right]\left\{\begin{array}{c} \partial \Theta \\ Z^{S}\\ \partial Z^{S} \end{array}\right\},$$

(25) $$\begin{array}{cc} \left\{\begin{array}{c} \langle Q\rangle \\ \langle Q\partial h^{A}\rangle \\ \langle Qh^{A}\rangle \\ \langle Qp^{R}\rangle \end{array}\right\} & =\left[\begin{array}{cccc} \langle kGA\rangle & \langle kGA\partial h^{B}\rangle & \langle kGAh^{B}\rangle & \langle kGAp^{S}\rangle \\ \langle kGA\partial h^{A}\rangle & \langle kGA\partial h^{A}\partial h^{B}\rangle & \langle kGA\partial h^{A}h^{B}\rangle & \langle kGA\partial h^{A}p^{S}\rangle \\ \langle kGAh^{A}\rangle & \langle kGAh^{A}\partial h^{B}\rangle & \langle kGAh^{A}h^{B}\rangle & \langle kGAh^{A}p^{S}\rangle \\ \langle kGAp^{R}\rangle & \langle kGA\partial h^{B}p^{R}\rangle & \langle kGAh^{B}p^{R}\rangle & \langle kGAp^{R}p^{S}\rangle \end{array}\right]\left\{\begin{array}{c} \left(\partial W-\Theta \right)\\ V^{B}\\ \partial V^{B}\\ -Z^{S} \end{array}\right\}. \end{array}$$

The 2(1+N) differential equations of the tolerance model for the macrodisplacements $W\left(\cdot \right)$, $\Theta \left(\cdot \right)$ and their fluctuation amplitudes $V^{A}\left(\cdot \right)$ and $Z^{R}\left(\cdot \right)$ can be now written as:

(26) $$\begin{array}{cc} \; & -\partial \langle Q\rangle +\langle \mu \rangle \ddot{W}+\langle \mu h^{A}\rangle {\ddot{V}}^{A}=0,\\ \; & -\partial \langle M\rangle -\langle Q\rangle +\langle \vartheta \rangle \ddot{\Theta }+\langle \vartheta p^{S}\rangle {\ddot{Z}}^{S}=0,\\ \; & \langle Q\partial h^{A}\rangle -\partial \langle Qh^{A}\rangle +\langle \mu h^{A}\rangle \ddot{W}+\langle \mu h^{A}h^{B}\rangle {\ddot{V}}^{B}=0,\\ \; & \langle M\partial p^{R}\rangle -\partial \langle Mp^{R}\rangle -\langle Qp^{R}\rangle +\langle \vartheta p^{R}\rangle \ddot{\Theta }+\langle \vartheta p^{R}p^{S}\rangle {\ddot{Z}}^{S}=0. \end{array}$$

Finally, a system of differential equations with constant coefficients is obtained. The number of these equations depends on the number of introduced fluctuation shape functions. Additionally, some of the coefficients depend on the size *l* of the periodicity cell. From the principle of stationary action, we can also conclude the natural boundary conditions:

(27) $${\left.\langle Q\rangle \delta W\right|}_{0}^{L}+{\left.\langle M\rangle \delta \Theta \right|}_{0}^{L}+{\left.\langle Qh^{A}\rangle \delta V^{A}\right|}_{0}^{L}+{\left.\langle Mp^{R}\rangle \delta Z^{R}\right|}_{0}^{L}=0.$$

Together with the averaged equation of motion, the following natural boundary conditions (for x=0,L) with averaged coefficients are obtained:

(28) $$\begin{array}{cc} \; & \langle Q\rangle =0\;\mathrm{or}\;W=0,\;\langle M\rangle =0\;\mathrm{or}\;\Theta =0,\\ \; & \langle Qh^{A}\rangle =0\;\mathrm{or}\;V^{A}=0,\;\langle Mp^{R}\rangle =0\;\mathrm{or}\;Z^{R}=0. \end{array}$$

### 4.2. Bernoulli Beam Tolerance Model

Let us now introduce tolerance weight-averaged bending moments for the Bernoulli beam model:

(29) $$\left\{\begin{array}{c} \langle M\rangle \\ \langle M\partial^{2}h^{A}\rangle \\ \langle M\partial h^{A}\rangle \\ \langle Mh^{A}\rangle \end{array}\right\}=\left[\begin{array}{cccc} \langle EJ\rangle & \langle EJ\partial^{2}h^{B}\rangle & \langle EJ\partial h^{B}\rangle & \langle EJh^{B}\rangle \\ \langle EJ\partial^{2}h^{A}\rangle & \langle EJ\partial^{2}h^{A}\partial^{2}h^{B}\rangle & \langle EJ\partial^{2}h^{A}\partial h^{B}\rangle & \langle EJ\partial^{2}h^{A}h^{B}\rangle \\ \langle EJ\partial h^{A}\rangle & \langle EJ\partial h^{A}\partial h^{B}\rangle & \langle EJ\partial h^{A}\partial h^{B}\rangle & \langle EJ\partial h^{A}h^{B}\rangle \\ \langle EJh^{A}\rangle & \langle EJh^{A}\partial^{2}h^{B}\rangle & \langle EJh^{A}\partial h^{B}\rangle & \langle EJh^{A}h^{B}\rangle \end{array}\right]\left\{\begin{array}{c} \partial^{2}W\\ V^{B}\\ 2\partial V^{B}\\ \partial^{2}V^{B} \end{array}\right\}.$$

This leads to a system of *N*+1 differential equations for macro-deflection and its fluctuation amplitudes:

(30) $$\begin{array}{cc} \; & \partial^{2}\langle M\rangle +\langle \mu \rangle \ddot{W}+\langle \mu h^{A}\rangle {\ddot{V}}^{A}=0,\\ \; & \langle M\partial^{2}h^{A}\rangle -2\partial \langle M\partial h^{A}\rangle +\partial^{2}\langle Mh^{A}\rangle +\langle \mu h^{A}\rangle \ddot{W}+\langle \mu h^{A}h^{B}\rangle {\ddot{V}}^{B}=0, \end{array}$$

and natural boundary conditions:

(31) $$-{\left.\partial \langle M\rangle \delta W\right|}_{0}^{L}+{\left.\langle M\rangle \partial \delta W\right|}_{0}^{L}+{\left.\langle Mh^{A}\rangle \partial \delta V^{A}\right|}_{0}^{L}+{\left.\left(\partial \langle Mh^{A}\rangle -2\langle M\partial h^{A}\rangle \right)\delta V^{A}\right|}_{0}^{L}=0,$$

where $W\left(x,\;t\right)$, $V^{A}\left(x,\;t\right)$ are the new kinematic unknowns. Together with the averaged equations of motion, the following natural boundary conditions (for x=0,L) with averaged coefficients are obtained:

(32) $$\begin{array}{cc} \; & -\partial \langle M\rangle =0\;\mathrm{or}\;W=0,\;\langle M\rangle =0\;\mathrm{or}\;\partial W=0,\\ \; & \partial \langle Mh^{A}\rangle -2\langle M\partial h^{A}\rangle =0\;\mathrm{or}\;V^{A}=0,\;\langle Mh^{A}\rangle =0\;\mathrm{or}\;\partial V^{A}=0. \end{array}$$

It is worth mentioning that expressions (28) and (32) reduce to classic natural boundary conditions for a homogeneous beam.

## 5. Applications

### 5.1. Problem Statement

We consider an elastic beam of length *L* with *l*-periodic variation of geometric and material properties. These two types of properties variation are: variable height $\delta \left(x+l\right)=\delta \left(x\right)$ and variable material properties $E\left(x+l\right)=E\left(x\right)$, $\rho \left(x+l\right)=\rho \left(x\right)$. The first case is related to a piecewise change of beam cross-section (stepped beam) for a beam made of a homogeneous material. Then, for steel the Young modulus $E_{s}=205\;\mathrm{GPa}$, the mass density $\rho_{s}=7850\;\mathrm{kg}/m^{3}$, the Poisson’s ratio $\nu_{s}=3/10$ are assumed. The cross section is rectangular: $b_{0}\times h_{0}=0.01\times 0.0250\;m$, $b_{1}\times h_{1}=0.01\times 0.0375\;m$.

In the second case, a beam consists of repetitive sections of aluminium and steel; therefore, different Young moduli and mass densities are considered (bi-material beam). For aluminium, the Young modulus $E_{a}=69\;\mathrm{GPa}$, the mass density $\rho_{a}=2700\;\mathrm{kg}/m^{3}$, the Poisson’s ratio $\nu_{a}=3/10$ are assumed. The cross section is rectangular: $b_{0}=b_{1}=0.01\;m$, $h_{0}=h_{1}=0.0250\;m$. The length of the beam for both cases, is L=1.0m. The shear coefficient was assumed equal to $k=\left(10+10\nu \right)/\left(12+11\nu \right)$.

**Figure 2:**
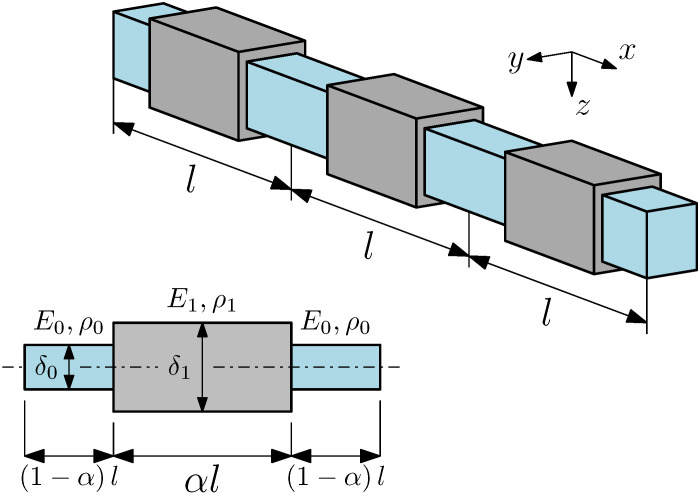
Geometry of the beam and the periodicity cell under consideration.

A fragment of the beam and a single periodicity cell are illustrated in Figure 2. In all of the cases, the length of the cell length was assumed to be equal; l=L/10. The analysis was performed for seven values of the saturation parameter α=1/8,1/4,…,3/4,7/8, which stands for the relative length of the central segment of the cell.

### 5.2. Effective Properties

**Figure 3:**
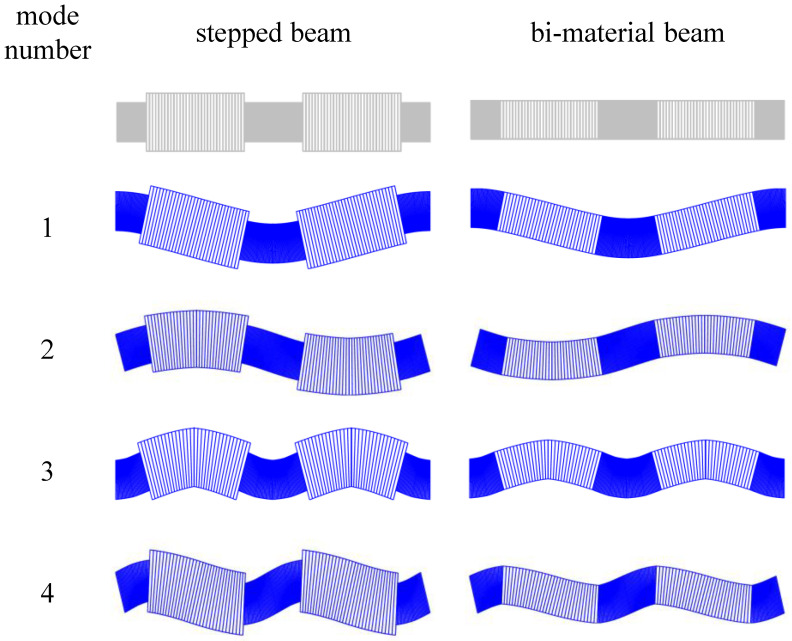
The first four eigenmodes for stepped and bi-material beams for α=5/8.

Determining of the fluctuation shape functions is a crucial point in calculation of the averaged coefficients of the Equations (26) and (30). These functions have to, at least approximately, represent the eigenmodes of a periodicity cell. From previous works, we concluded that the best results were obtained when analysing a combination of two cells. A finite element model of a two-cell assembly with periodic boundary conditions was used to obtain the eigenmodes. The minimal number of elements per unit cell was set by a prior convergence study. The first four of the fluctuation shape functions $h^{A}$ for stepped and bi-material beam are displayed in Figure 3.

### 5.3. Natural Vibrations Analysis

In order to obtain a system of algebraic frequency equations, the Galerkin method is applied. The trial functions are assumed in the form of truncated trigonometric series. In the case of a Timoshenko beam, these are:

(33) $$\begin{array}{cc} W\left(x,t\right) & =\sum_{m=1}^{M_{w}}W_{m}X_{m}^{W}\left(x\right)\cos\left(\omega t\right),\;\Theta \left(x,t\right)=\sum_{m=1}^{M_{\Theta }}\Theta_{m}X_{m}^{\Theta }\left(x\right)\cos\left(\omega t\right),\\ V^{A}\left(x,t\right) & =\sum_{n=1}^{M_{V^{A}}}V_{m}^{A}X_{m}^{V^{A}}\left(x\right)\cos\left(\omega t\right),\;Z^{R}\left(x,t\right)=\sum_{n=1}^{M_{Z^{R}}}Z_{m}^{R}X_{m}^{Z^{R}}\left(x\right)\cos\left(\omega t\right), \end{array}$$

where the *x*-dependent functions $X_{m}$ and $Y_{m}$ have to satisfy the appropriate boundary conditions. The macro-displacements solutions for a simply supported beam were assumed as $X_{m}\left(x\right)=\sin\left(m\pi x/L\right)$ and $Y_{m}\left(x\right)=\cos\left(m\pi x/L\right)$. For the fluctuation amplitudes $V^{A}$ and $Z^{A}$, which come in pairs, one has to carefully examine the boundary conditions. The fluctuation shape functions for a symmetric unit cell are of two kinds.

If the fluctuation shape function $h^{A}$ is symmetric, its corresponding $p^{A}$ function is antisymmetric with respect to the centre of the cell, and vice versa. In that case, $X_{m}^{V^{A}}\left(x\right)=X_{m}^{Z^{A}}\left(x\right)=\sin\left(m\pi x/L\right)$; and in the opposite case, $X_{m}^{V^{A}}\left(x\right)=X_{m}^{Z^{A}}\left(x\right)=\cos\left[\left(m-1\right)\pi x/L\right]$. In case of the Euler–Bernoulli theory, the rotation of cross section is dependent of the deflection, and naturally unknowns $\Theta \left(x,t\right)$ and $Z^{R}\left(x,t\right)$ drop out of the equations.

Inserting the assumed solutions to either the Timoshenko (26) or Euler–Bernoulli (30) beam equations, leads to a homogeneous matrix equation. The non-trivial solutions for the eigenfrequencies ω and the eigenvectors are obtained through equating the determinant $\left|K_{\left[n\times n\right]}-\omega^{2}M_{\left[n\times n\right]}\right|$ to zero. The number of degrees of freedom is equal to $n=M_{w}+M_{\Theta }+N\left(M_{V^{A}}+M_{Z^{A}}\right)$ for the Timoshenko beam model and $n=M_{w}+NM_{V^{A}}$ for the Euler–Bernoulli model.

### 5.4. Exact Models and Their Numerical Solutions

Let us divide the beam into *n* sections wit constant geometrical and material properties, so that EJ=const, kGA=const, μ=const, ϑ=const. Then, in every section, the equations of motion according to the Timoshenko theory have the form:

(34) $$\begin{array}{cc} \; & kGA\left(\partial^{2}w-\partial \theta \right)-\mu \ddot{w}=0,\\ \; & EJ\partial^{2}\theta +kGA\left(\partial w-\theta \right)-\vartheta \ddot{\theta }=0. \end{array}$$

Now, we introduce the nondimensional coordinate $\xi_{i}=\frac{x_{i}}{l_{i}}$ for *i*-th segment, i=1,…,n. The relation between derivatives can be written as:

(35) $$\frac{\partial^{\left(n\right)}f\left(x_{i}\right)}{{\left(\partial x_{i}\right)}^{n}}={\left(\frac{1}{l_{i}}\right)}^{n}\frac{\partial^{\left(n\right)}f\left(\xi_{i}\right)}{{\left(\partial \xi_{i}\right)}^{n}}.$$

Solutions to the obtained differential equations can be sought in the form:

(36) $$\begin{array}{cc} w_{i}\left(\xi_{i}\right) & =C_{i1}\sin\left(a_{i}\xi_{i}\right)+C_{i2}\cos\left(a_{i}\xi_{i}\right)+C_{i3}\sinh\left(b_{i}\xi_{i}\right)+C_{i4}\cosh\left(b_{i}\xi_{i}\right),\\ \theta_{i}\left(\xi_{i}\right) & =\frac{\lambda_{i}^{4}s_{i}^{2}-a_{i}^{2}}{a_{i}l_{i}}C_{i2}\sin\left(a_{i}\xi_{i}\right)-\frac{\lambda_{i}^{4}s_{i}^{2}-a_{i}^{2}}{a_{i}l_{i}}C_{i1}\cos\left(a_{i}\xi_{i}\right)+\\ \; & +\frac{\lambda_{i}^{4}s_{i}^{2}+b_{i}^{2}}{b_{i}l_{i}}C_{i4}\sinh\left(b_{i}\xi_{i}\right)+\frac{\lambda_{i}^{4}s_{i}^{2}+b_{i}^{2}}{b_{i}l_{i}}C_{i3}\cosh\left(b_{i}\xi_{i}\right), \end{array}$$

where the coefficients are as follows:

(37) $$a_{i}^{2}=\frac{H_{i}+\sqrt{H_{i}^{2}-4K_{i}}}{2},\;b_{i}^{2}=\frac{-H_{i}+\sqrt{H_{i}^{2}-4K_{i}}}{2},$$

(38) $$\lambda_{i}^{4}=\omega^{2}\frac{\mu_{i}l_{i}^{4}}{E_{i}J_{i}},\;r_{i}^{2}=\frac{J_{i}}{A_{i}l_{i}^{2}},\;s_{i}^{2}=\frac{E_{i}J_{i}}{k_{i}G_{i}A_{i}l_{i}^{2}},$$

(39) $$H_{i}=\lambda_{i}^{4}\left(r_{i}^{2}+s_{i}^{2}\right),\;K_{i}=\lambda_{i}^{4}\left(\lambda_{i}^{4}r_{i}^{2}s_{i}^{2}-1\right).$$

The internal forces in *i*-th section of the beam are equal to:

(40) $$\begin{array}{ccc} M_{i}\left(\xi_{i}\right) & =-\frac{E_{i}J_{i}}{l_{i}}\frac{\partial \theta_{i}\left(\xi_{i}\right)}{\partial \xi_{i}},\;Q_{i}\left(\xi_{i}\right) & =k_{i}G_{i}A_{i}\left(\frac{1}{l_{i}}\frac{\partial w_{i}\left(\xi_{i}\right)}{\partial \xi_{i}}-\theta_{i}\left(\xi_{i}\right)\right). \end{array}$$

Now, one has to define the continuity conditions of displacements and internal forces on the interfaces between the neighbouring sections:

(41) $$\begin{array}{cc} \; & w_{i}\left(\xi_{i}=0\right)=w_{i-1}\left(\xi_{i-1}=1\right),\;\theta_{i}\left(\xi_{i}=0\right)=\theta_{i-1}\left(\xi_{i-1}=1\right),\\ \; & M_{i}\left(\xi_{i}=0\right)=M_{i-1}\left(\xi_{i-1}=1\right),\;Q_{i}\left(\xi_{i}=0\right)=Q_{i-1}\left(\xi_{i-1}=1\right). \end{array}$$

Moreover, the boundary conditions have to be defined on the beam ends. For a simply supported beam these conditions are:

(42) $$w_{1}\left(\xi_{1}=0\right)=w_{n}\left(\xi_{n}=1\right)=0,\;M_{1}\left(\xi_{1}=0\right)=M_{n}\left(\xi_{n}=1\right)=0.$$

From the above relations, we obtain a set of homogeneous algebraic equations for the constants $C_{11}-C_{n4}$:

(43) A(ω)C=0.

The condition for existence of nontrivial solutions is as follows:

(44) det(A(ω))=0,

from which we obtain a transcendental equation, which was solved in two steps. First, the roots were isolated by linear search. Next, the secant method was applied to determine the roots with precision $\Delta =1-\omega_{i}/\omega_{i-1}<10^{-30}$.

The solution procedure has to be simplified in order to analyse this problem in the framework of Bernoulli theory. Assuming that the shear angle γ is now equal to zero, the average cross-section rotation θ=∂w, we obtain Equation (7), bearing in mind that now the bending stiffness is constant. The solution for the deflection *w* given by first of the expressions in Equation (36) is still valid. Now, in the expressions (38) and (39) $r_{i}^{2}\ll 1$, $s_{i}^{2}\ll 1$$H_{i}\approx 0$, $K_{i}\approx -\lambda_{i}^{4}$. The coefficients in Equation (37) are now equal, $a_{i}^{2}=b_{i}^{2}=\lambda_{i}^{2}$. The boundary and continuity conditions remain the same, but the bending moment and shear force in Equation (40) have to be written as:

(45) $$\begin{array}{ccc} M_{i}\left(\xi_{i}\right) & =-\frac{E_{i}J_{i}}{l_{i}^{2}}\frac{\partial^{2}w_{i}\left(\xi_{i}\right)}{\partial \xi_{i}^{2}},\;Q_{i}\left(\xi_{i}\right) & =-\frac{E_{i}J_{i}}{l_{i}^{3}}\frac{\partial^{3}w_{i}\left(\xi_{i}\right)}{\partial \xi_{i}^{3}}, \end{array}$$

and the general form of Equations (43) and (44) remains unchanged.

## 6. Results and Discussion

In this section, a numerical study of natural frequencies of considered Bernoulli and Timoshenko beams will be carried out. The influence of beam stiffness and mass distribution will be investigated in relation to the saturation parameter α. First, low order frequencies $\omega_{1}$–$\omega_{5}$ for the two considered cases, for various saturation parameter α=1/2, according to the proposed and the exact models, are investigated.

The results are displayed in Table 1. The discrepancies between the averaged and exact models increase slowly with increasing mode number, and the agreement is remarkable. The relative differences are less than 0.0075%.

Then, frequencies $\omega_{1}$–$\omega_{5}$ for the two considered cases, for various values of saturation parameter α, according to both models, are investigated. The results are displayed in Table 2 and Table 3 for a stepped beam according to the Timoshenko and Bernoulli theories, respectively. The results for a bi-material beam are shown in Table 4 and Table 5. The results of the TA and FE models are in excellent agreement, as the relative difference between these models does not exceed 0.02%.

It can be seen that the low frequencies increase with increasing α parameter value for a stepped beam. In the case of a bi-material beam, these frequencies are lowest for α=1/2 and increase when this parameter decreases towards its minimum value and when it increases towards its maximum value. For these low frequencies, the relative difference between Bernoulli and Timoshenko model is less than 5% for the stepped beam and less than 3% for the bi-material beam.

**Figure 4:**
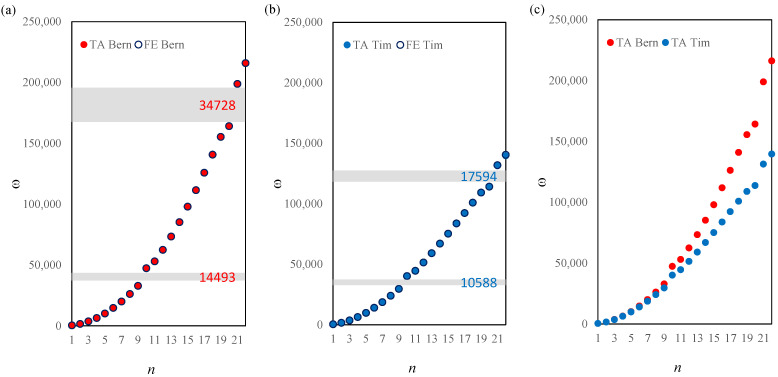
Comparison of the natural frequencies between the TA and FE models according to the Bernoulli (**a**) and Timoshenko (**b**) theories, and comparison between the two models (**c**), α=1/2 for a bi-material beam.

The frequency spectrum for a stepped beam is presented in Figure 4 for Bernoulli and Timoshenko beam models for the saturation parameter α=1/2. The first frequency gap is placed between the 9th and 10th natural frequency and is equal to Δω=14,493rad/s according to the Bernoulli beam model and Δω=10,588rad/s according to the Timoshenko beam model. The second frequency gap can be found between the 20th and 21st natural frequency and is equal to Δω=34,728rad/s for the Bernoulli model and Δω=17,594rad/s for the Timoshenko model.

**Figure 5:**
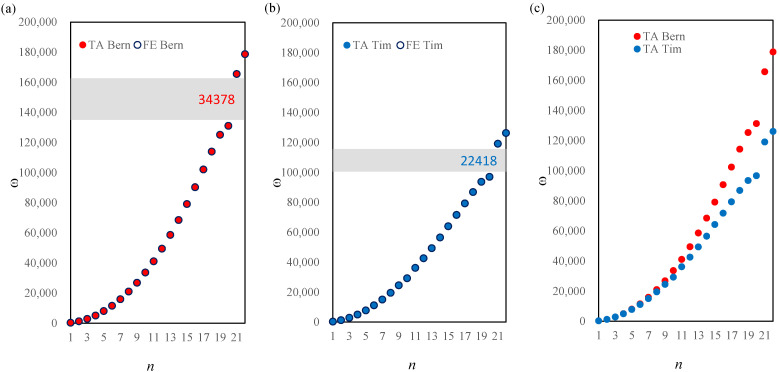
Comparison of the natural frequencies between the TA and FE models according to the Bernoulli (**a**) and Timoshenko (**b**) theories, and comparison between the two models (**c**), α=1/2 for a stepped beam.

Similar results are presented in Figure 5 for a bi-material beam and for the saturation parameter value α=1/2. There is no distinct frequency gap near the 10th frequency for either of the models. The difference between the 20th and 21st frequency is equal to Δω=34,378rad/s and Δω=22,418rad/s for the Bernoulli and Timoshenko model, respectively.

**Figure 6:**
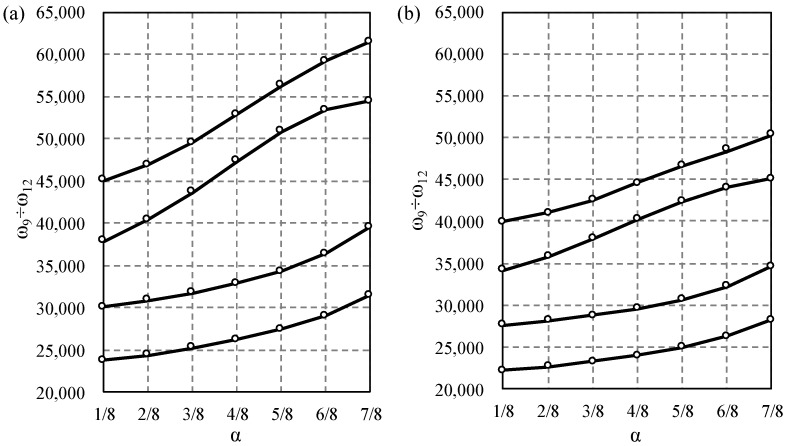
Comparison of the 8th to 11th eigenfrequency for stepped beams according to the Bernoulli (**a**) and Timoshenko (**b**) theories, in relation to the saturation parameter α. TA—solid line, and FE—dots.

**Figure 7:**
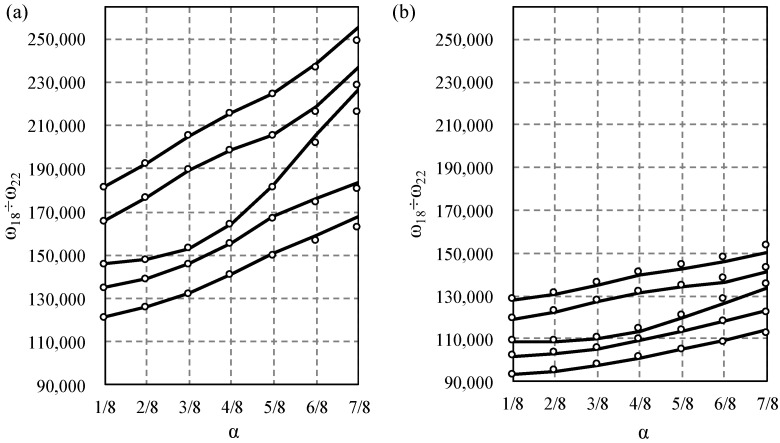
Comparison of the 18th to 22nd eigenfrequency for stepped beams according to the Bernoulli (**a**) and Timoshenko (**b**) theories, in relation to the saturation parameter α. TA—solid line, and FE—dots.

**Figure 8:**
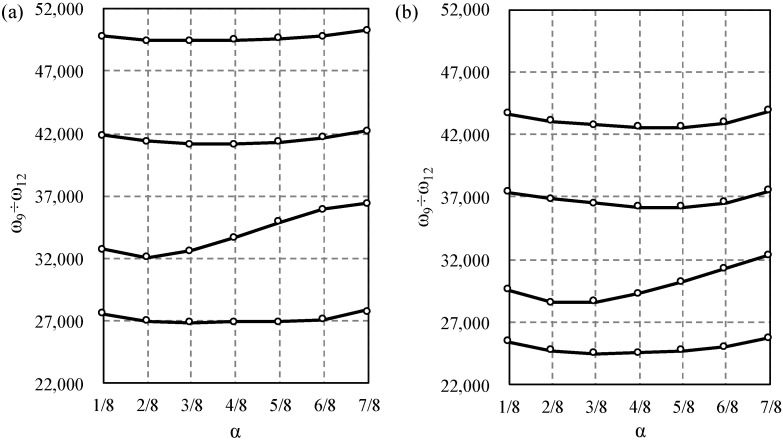
Comparison of the 8th to 11th eigenfrequency for bi-material beams according to the Bernoulli (**a**) and Timoshenko (**b**) theories, in relation to the saturation parameter α. TA—solid line, and FE—dots.

**Figure 9:**
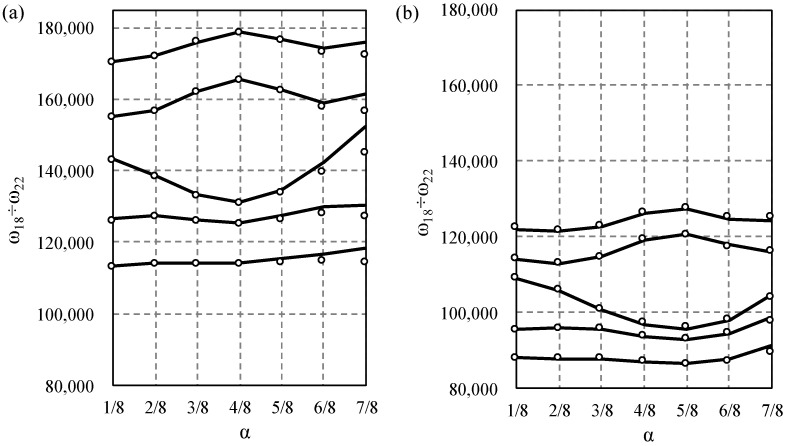
Comparison of the 18th to 22nd eigenfrequency for bi-material beams according to the Bernoulli (**a**) and Timoshenko (**b**) theories, in relation to the saturation parameter α. TA—solid line, and FE—dots.

Next, the influence of the saturation parameter α on width of the first and second frequency gap was studied and displayed in Figure 6, Figure 7, Figure 8 and Figure 9. The results for the Bernoulli and Timoshenko averaged models are compared with finite element results.

For the stepped beam case, the maximum difference occurs between the 9th and 10th frequency for α=3/4 for both models, Figure 6. For higher frequencies, the maximum difference occurs between the 19th and 20th frequency for α=7/8 for the Bernoulli model; however, according to the Timoshenko model, it is between the 20th and 21st frequency for α=1/2, cf. Figure 7.

For the bi-material beam case in the lower frequency spectrum, the largest gap occurs between 10th and 11th frequency for α=1/4 for both models, Figure 8, and there is no distinct gap near α=1/4, which confirms the data in Figure 6. For higher frequencies, the maximum difference occurs between the 20th and 21st for α=1/2 according to the Bernoulli model, and for α=5/8 according to the Timoshenko model, cf. Figure 9.

The general conclusion that can been formulated from the considerations is that the frequencies obtained from the Timoshenko beam model are lower then the calculated from the Bernoulli model, and the differences increase with increasing the number of eigenmode half-waves.

Considering the differences between the results obtained from the compared theories, let us introduce the relative differences $r_{n}=\left(\omega_{B}^{\left(n\right)}-\omega_{T}^{\left(n\right)}\right)/\omega_{T}^{\left(n\right)}$, corresponding to the frequency number *n*.

The growth of these differences, as a function of the frequency number, is approximately quadratic; at the same time, it is nearly linear as a function of frequency. This can be said about both the stepped and bi-material beam cases. For example, the relative difference for the stepped beam varies from $r_{1}=0.1\%$ for α=1/8 and $r_{1}=0.2\%$ for α=7/8 to $r_{21}=39.3\%$ for α=1/8 and $r_{21}=67.7\%$ for α=7/8. In the case of a bi+material beam, these values vary from $r_{1}=0.1\%$ for all values of α to $r_{19}=31.9\%$ for α=1/8 and $r_{19}=45.8\%$ for α=7/8. The situation is therefore, unsurprisingly, similar to that of uniform beams of constant height.

On the other hand, it is expected that the heterogeneity at the cell level should have some impact on the results. With fixed proportions of cell sections’ stiffness, the parameter α should be the crucial parameter. Let us first analyse the results for the stepped beam.

When increasing this parameter from its lowest to the highest value, the relative differences increase as well. However, up to the fifth form of vibration, these differences do not exceed 5%. For the sixth form, these values range from $r_{6}=3.8\%$ for α=1/8 to $r_{6}=6.9\%$ for α=7/8. For the 10th mode, the differences range from 11% to 21%. Generally, for the higher frequencies, the relative frequencies increase faster.

For the bi-material beam, the distribution of differences is of a slightly different nature. Initially, as in the previous case, these differences are less than 5% for the first five modes of vibration. Up to the eighth frequency, the discrepancies do not exceed 8% and are basically independent of the parameter α. For higher modes, the relative differences tend to grow from their minimal values for extreme values of α to their maximal values for intermediate values of this parameter. However, the changes in the discrepancies are much more moderate than for the stepped beam. For the 11th mode, they are equal to $r_{11}=14.2\%$ for α=1/8 through $r_{11}=16.7\%$ for α=5/8 and to $r_{11}=14.7\%$ for α=7/8.

**Figure 10:**
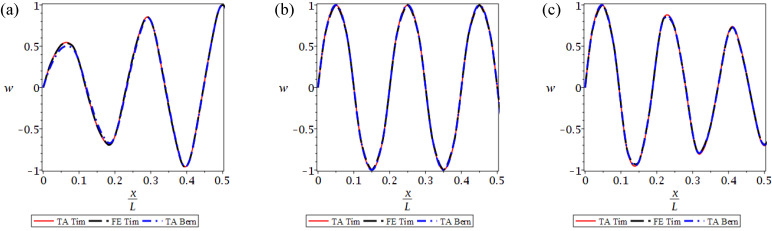
The 9th (**a**), 10th (**b**) and 11th (**c**) natural vibration modes presented for the left half of the beam, for a stepped beam for the TA and FE Timoshenko beam models and for the TA Bernoulli model.

**Figure 11:**
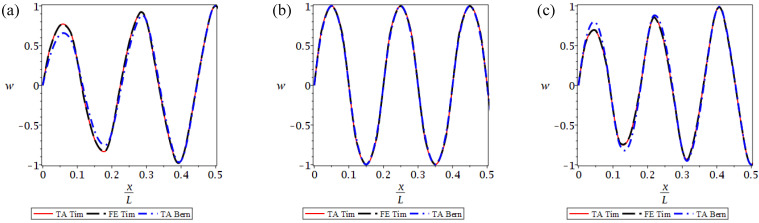
The 9th (**a**), 10th (**b**) and 11th (**c**) natural vibration modes presented for the left half of the beam, for a bi-material beam for the TA and FE Timoshenko beam models and for the TA Bernoulli model.

In Figure 10 and Figure 11 the 9th, 10th and 11th mode shapes of the considered beams are displayed. The shapes are shown for the left half of the beam, $x\in \left(0,L/2\right)$ for the stepped beam (Figure 10) and for the bi-material beam (Figure 11). It can be seen that the TA and FE results are in good agreement, but the deflections obtained from the Bernoulli beam model differ slightly for some cases, especially near the beam supports, as the shear forces are maximal there.

## 7. Conclusions

In this paper, the basic dynamic properties of beams with periodically varying geometric and material properties were investigated. The periodic variation of geometric and material properties produces the band gaps in the frequency domain. It was shown by comparison with FE results that the proposed tolerance-averaging technique for differential operators leads to reliable homogenized models of beams.

As some of the averaged coefficients of obtained differential equations depend explicitly on the unit cell length, the positions of gaps in the frequency spectrum were determined properly. They can also be predicted by considering the order of the vibrational forms of the periodicity cell. We confirmed that, for the considered boundary conditions, the trial functions used in the Galerkin method solutions can be adopted from solutions of a homogeneous beam.

As the second result, we demonstrated, through comparison between both models, that taking the Euler–Bernoulli assumptions should be considered with caution. In the higher frequency range, the simplification of neglecting the shear susceptibility and rotational inertia terms leads to unacceptable discrepancies in relation to the Timoshenko model, which was proven by many researchers to be more accurate.

Applications of the so-called technical theories of beams have their limitations. The Bernoulli model’s application possibilities are limited to the analysis of rather slender beams. More precisely, it is required that the half-wave length of any eigenmode is significantly greater than the cross-section height. The Timoshenko model, concerning shear deformability and rotatory inertia as well, has the potential to yield correct results for thick beams.

Let us be concerned only with the technical 1-D theories of continuous beams here. The main factors that matter are the half-wave length of the *n*-th vibration mode $\lambda_{n}=L/n$ and the length-to-height ratio of the beam η=L/h. It was shown in [51] that the possible scope of application of the Timoshenko model is rather significant.

Comparing the results obtained from this model and a 2-D FE model, the half-wave length can be of the order of the beam thickness. Thus, the number of eigenfrequencies and eigenmodes is limited by the ratio of the mode half-wave length to the beam thickness. It was assumed here that the safe range of applicability of Timoshenko’s theory is for the ratio $\lambda /h_{\max}$ greater than 1.25.

A shortcoming of this paper is the modelling assumption that the bonds between neighbouring sections of the beam are ideal. Especially in stepped beams, there are stress-free areas of the beam adjacent to the height jump. Therefore, bending stresses do not occur over the entire height of the deeper section. This could be taken into account by assuming a functional change in the cross-section height in the vicinity of these points of transition between the two sections. The proposed models allow for the introduction of such an assumption. However, a significant qualitative change in the results is not expected.

## Figures and Tables

**Table 1:** Comparison of the first five natural frequencies according to both beam theories for stepped and bi-material beams, α=1/2: TA and exact results.

Mode	Stepped Beam				Bi-Material Beam			
	Timoshenko		Bernoulli		Timoshenko		Bernoulli	
	Exact	TA	Exact	TA	Exact	TA	Exact	TA
1	403.881	403.883	404.479	404.479	314.946	314.947	315.280	315.280
2	1609.50	1609.52	1619.02	1619.02	1257.70	1257.71	1263.06	1263.06
3	3599.16	3599.29	3647.04	3647.06	2822.20	2822.27	2849.48	2849.49
4	6344.62	6345.06	6494.45	6494.58	4998.94	4999.17	5085.78	5085.83
5	9808.91	9809.51	10,169.4	10,169.9	7775.64	7776.22	7989.56	7989.76

**Table 2:** Comparison of the first five natural frequencies according to the Timoshenko beam theory for a stepped beam: TA and FE results.

Model	n	α						
		1/8	1/4	3/8	1/2	5/8	3/4	7/8
TA	1	369.347	377.603	388.859	403.883	423.904	450.980	488.791
	2	1472.47	1505.27	1550.00	1609.52	1688.57	1795.18	1943.82
	3	3294.91	3367.92	3467.49	3599.29	3773.35	4007.12	4332.22
	4	5813.56	5941.46	6115.87	6345.06	6645.39	7046.58	7602.84
	5	8997.96	9194.10	9461.46	9809.51	10,261.3	10,861.2	11,690.7
FE	1	369.347	377.602	388.858	403.881	423.904	450.980	488.792
	2	1472.47	1505.27	1549.98	1609.50	1688.56	1795.18	1943.83
	3	3294.93	3367.91	3467.39	3599.19	3773.33	4007.17	4332.37
	4	5813.66	5941.45	6115.56	6344.75	6645.39	7046.82	7603.61
	5	8998.31	9194.17	9460.76	9808.82	10,261.3	10,861.9	11,693.3

**Table 3:** Comparison of the first five natural frequencies according to teh Bernoulli beam theory for a stepped beam: TA and FE results.

Model	n	α						
		1/8	1/4	3/8	1/2	5/8	3/4	7/8
TA	1	369.774	378.077	389.388	404.479	424.590	451.788	489.782
	2	1479.26	1512.81	1558.42	1619.02	1699.48	1808.02	1959.50
	3	3328.95	3405.79	3509.79	3647.06	3828.15	4071.34	4410.29
	4	5919.73	6059.78	6248.22	6494.58	6816.58	7246.27	7844.05
	5	9252.89	9478.83	9780.50	10,169.9	10,672.9	11,338.7	12,263.1
FE	1	369.774	378.077	389.388	404.479	424.590	451.788	489.781
	2	1479.26	1512.81	1558.42	1619.02	1699.48	1808.01	1959.50
	3	3328.95	3405.79	3509.78	3647.04	3828.11	4071.28	4410.25
	4	5919.73	6059.77	6248.18	6494.45	6816.31	7245.90	7843.80
	5	9252.89	9478.80	9780.33	10,169.4	10,671.8	11,337.1	12,262.1

**Table 4:** Comparison of the first five natural frequencies according to the Timoshenko beam theory for a bi-material beam: TA and FE results.

Model	n	α						
		1/8	1/4	3/8	1/2	5/8	3/4	7/8
TA	1	337.554	324.170	317.013	314.947	317.673	325.588	339.966
	2	1346.40	1293.75	1265.76	1257.71	1268.39	1299.42	1356.03
	3	3015.30	2900.14	2839.54	2822.27	2845.45	2912.89	3036.93
	4	5326.13	5129.46	5027.74	4999.17	5038.31	5152.30	5364.56
	5	8254.66	7963.10	7816.31	7776.22	7833.30	7999.58	8314.94
FE	1	337.554	324.169	317.011	314.946	317.672	325.588	339.966
	2	1346.40	1293.74	1265.74	1257.70	1268.39	1299.42	1356.04
	3	3015.31	2900.08	2839.43	2822.21	2845.44	2912.91	3036.98
	4	5326.19	5129.31	5027.41	4999.02	5038.30	5152.3	5364.83
	5	8254.87	7962.80	7815.60	7775.93	7833.35	7999.91	8315.94

**Table 5:** Comparison of the first five natural frequencies according to the Bernoulli beam theory for a bi-material beam: TA and FE results.

Model	n	α						
		1/8	1/4	3/8	1/2	5/8	3/4	7/8
TA	1	337.911	324.513	317.347	315.280	318.009	325.933	340.326
	2	1352.10	1299.24	1271.13	1263.06	1273.79	1304.93	1361.77
	3	3043.96	2927.88	2866.82	2849.49	2872.86	2940.78	3065.77
	4	5415.97	5217.14	5114.44	5085.83	5125.34	5240.32	5454.90
	5	8471.71	8177.14	8029.54	7989.76	8046.98	8213.98	8532.96
FE	1	337.911	324.513	317.347	315.280	318.009	325.933	340.326
	2	1352.10	1299.24	1271.13	1263.06	1273.78	1304.93	1361.77
	3	3043.96	2927.88	2866.82	2849.48	2872.84	2940.76	3065.76
	4	5415.97	5217.13	5114.43	5085.78	5125.24	5240.17	5454.81
	5	8471.71	8177.12	8029.48	7989.57	8046.58	8213.42	8532.61

## Data Availability

The data presented in this study are available on request from the corresponding author.

## Abbreviations

The following abbreviations are used in this manuscript:

TA	Tolerance Averaging
FE	Finite Element
